# Force Fields, Quantum-Mechanical- and Molecular-Dynamics-Based Descriptors of Radiometal–Chelator Complexes

**DOI:** 10.3390/molecules29184416

**Published:** 2024-09-17

**Authors:** Işılay Öztürk, Silvia Gervasoni, Camilla Guccione, Andrea Bosin, Attilio Vittorio Vargiu, Paolo Ruggerone, Giuliano Malloci

**Affiliations:** Department of Physics, University of Cagliari, I-09042 Monserrato (CA), Italy; isilay.ozturk@dsf.unica.it (I.Ö.); camilla.guccione@dsf.unica.it (C.G.); andrea.bosin@dsf.unica.it (A.B.); attilio.vargiu@dsf.unica.it (A.V.V.); paolo.ruggerone@dsf.unica.it (P.R.)

**Keywords:** metal-based radiopharmaceuticals, functional chelators, molecular databases, all-atom force fields, quantum mechanics, density functional theory, molecular dynamics simulations

## Abstract

Radiopharmaceuticals are currently a key tool in cancer diagnosis and therapy. Metal-based radiopharmaceuticals are characterized by a radiometal–chelator moiety linked to a bio-vector that binds the biological target (e.g., a protein overexpressed in a particular tumor). The right match between radiometal and chelator influences the stability of the complex and the drug’s efficacy. Therefore, the coupling of the radioactive element to the correct chelator requires consideration of several features of the radiometal, such as its oxidation state, ionic radius, and coordination geometry. In this work, we systematically investigated about 120 radiometal–chelator complexes taken from the Cambridge Structural Database. We considered 25 radiometals and about 30 chelators, featuring both cyclic and acyclic geometries. We used quantum mechanics methods at the density functional theoretical level to generate the general AMBER force field parameters and to perform 1 µs-long all-atom molecular dynamics simulations in explicit water solution. From these calculations, we extracted several key molecular descriptors accounting for both electronic- and dynamical-based properties. The whole workflow was carefully validated, and selected test-cases were investigated in detail. Molecular descriptors and force field parameters for the complexes considered in this study are made freely available, thus enabling their use in predictive models, molecular modelling, and molecular dynamics investigations of the interaction of compounds with macromolecular targets. Our work provides new insights in understanding the properties of radiometal–chelator complexes, with a direct impact for rational drug design of this important class of drugs.

## 1. Introduction

Radiopharmaceuticals are used to treat several cancer types and neuroendocrine disorders (e.g., prostate and thyroid cancer) thanks to their ability to specifically target the damaged tissues [1,2,3,4,5]. Metal-based radiopharmaceuticals are generally constituted by a polydentate ligand (functional chelator) that stably coordinates a radiometal, which is connected to a bio-vector that binds the target protein. As a result, a specific radiometal with diagnostic or therapeutic properties can reach the desired molecular target within the body. A prominent example of marketed drugs from this class is represented by peptide-based radiopharmaceuticals targeting somatostatin receptors for the treatment of neuroendocrine tumors [6,7].

**Figure 1:**
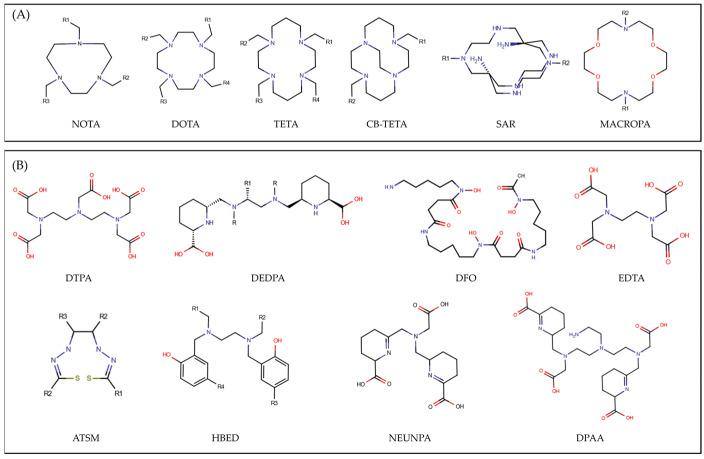
2D sketch of the (**A**) six main cyclic and (**B**) eight main acyclic chelator families considered in this study. For the IUPAC name of each family see Supplementary Materials.

To determine the diagnostic or therapeutic function of specific radiometals, several key parameters should be considered, such as ionic radius, charge state, coordination number and geometry, and specific decay properties [8,9,10]. Bi^3+^ (Bismuth), Pb^2+^ (Lead), Sb^3+^ (Antimony), Y^3+^ (Yttrium), and Ac^3+^ (Actinium) are used in targeted radiotherapy due to their alpha and beta-emitting properties. In contrast, metals like Cu^2+^ (Copper), Ga^3+^ (Gallium), In^3+^ (Indium), and Zr^4+^ (Zirconium) are employed for imaging purposes, particularly in positron emission tomography (PET) and single photon emission computed tomography (SPECT), because of their positron and gamma emissions. Generally used for both diagnostic and therapeutic purposes are Co^2+^ (Cobalt) and Sc^3+^ (Scandium) [11,12]. Commonly employed chelators are characterized by different geometries, such as cyclic and acyclic, and have been designed to create stable complexes with certain radiometals (Figure 1) [4,13,14]. Each class has distinct benefits [3,4,9,13,14]. Cyclic chelators are more rigid than their acyclic counterparts due to their restricted number of possible metal coordination sites. The disadvantage of cyclic scaffolds is slow binding kinetics that necessitate high temperatures and long waiting periods for radiolabelling. Conversely, acyclic ligands offer more flexibility, providing a greater number of potential metal coordination options, thus exhibiting rapid radiolabeling at ambient temperature. The drawback is a higher entropic cost, which results in a greater probability of decomplexation in vivo, as compared to cyclic chelators. Intense research in the field of chelator development has proven successful in mitigating the disadvantages of each class. As prominent examples, DOTA and NOTA are renowned for their exceptional stability with a wide range of radiometals, being, thus, widely used in PET and SPECT imaging [7,15]. Similarly, DTPA and EDTA are widely used acyclic chelators known for their ability to form strong complexes with various metal ions, finding numerous industrial and theranostic applications [16,17]. Matching chelators to radiometals for developing stable complexes is critical for successful radiopharmaceutical development and application in nuclear medicine [4]. However, no single chelator is universally effective for all metals, and in vivo stability of the whole radiopharmaceutical, in terms of biodistribution and clearance, plays a significant role as well [18,19]. Furthermore, chelators should block transchelation to prevent radiometal dissociation or binding to other molecules inside the body [4,15,17,20,21,22]. Therefore, finding suitable chelators capable of effectively binding to both the radiometal and the bio-vector is imperative for achieving the optimal ratio of radiopharmaceutical uptake between target and non-target tissues, whether for diagnostic imaging or therapeutic purposes.

Computational studies are currently used in a systematic way to provide molecular descriptors at different levels of accuracy and force field development [23,24,25,26]. In nuclear medicine, molecular simulations are a valuable tool for exploring whether a chelator is suitable for coordinating a radiometal and providing intrinsic physicochemical properties. In a recent investigation on radiopharmaceuticals binding somatostatin receptor 2, we noticed that altering the radiometal while maintaining the same chelator influenced how the compounds interact with the target protein. Furthermore, we found that changing the chelator while keeping the same metal also affects the site of specific interactions with the protein [6]. This suggested that specific radiometal–chelator pairing also influences the interaction with the biological target. However, from a computational point of view, the parametrization of radiometals is a well-known challenging task, due to the peculiar properties of these atoms (i.e., atomic orbitals with high angular momenta, multiple oxidation states, electronic state degeneracy, complicated chemical bonding, and multiple coordination numbers) [27,28].

To contribute to these lines of research, in this work, we collected a dataset of about 30 chelators and 25 radiometals, resulting in about 120 complexes, ranging in size from ~30 to ~90 atoms. Through a validated computational pipeline, for each complex we performed both quantum mechanics (QM) and molecular dynamics (MD) simulations to compute several key molecular descriptors (e.g., accounting for chemical stability, solvation effect, H-bonding properties, and flexibility). The radiometals and chelators considered and their usage for imaging or therapy purposes are reported in Table S1 of the Supplementary Materials. The complete set of compounds investigated with the corresponding chelator family are reported in Table S2. A schematic representation of both the cyclic and acyclic chelators that were considered is shown in Figure 1. For each complex, the molecular descriptors computed at the QM and MD levels are reported (see Table S3 for a detailed definition of each molecular descriptor). We additionally provided general AMBER force field (GAFF2) parameters that are made freely available and can be used for further computational studies.

## 2. Results and Discussion

In the following, we present the sample of compounds included in the database (Section 2.1) and the overall dataset structure (Section 2.2). Then, we report a characterization of radiometal–chelator complexes based on a sample of molecular descriptors derived from both QM and MD simulations (Section 2.3). Compounds bearing a cyclic or an acyclic chelator will be discussed separately, thus enabling a molecular properties comparison for the same chelator carrying different metals.

### 2.1. Sample of Compounds

**Figure 2:**
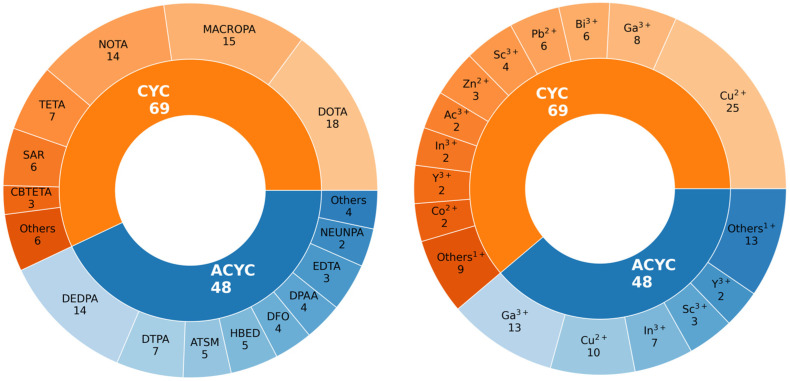
Graphical representation of the sample of compounds considered in this work (See Tables S1 and S2 for the full list of radiometal–chelator complexes and Figure 1 for a 2D sketch of the main families of CYC and ACYC compounds).

Figure 2 provides a graphical representation of the sample of radiometal–chelator complexes considered in this work. The study encompasses 117 compounds, listed in Table S2, broadly classified based on the chelator structure as cyclic (CYC) and acyclic (ACYC). Among these, 69 are CYC species, including 20 different radiometals within their structures, while the ACYC ones consist of 48 compounds incorporating 15 different radiometals. CYC molecules, characterized by their ring-like structure, are partitioned into 6 main families, namely DOTA, NOTA, TETA, SAR, CBTETA, and MACROPA (Figure 1A). Similarly, ACYC species are grouped into 8 main families: DTPA, DEDPA, DFO, EDTA, ATSM, HBED, NEUNPAA, and DPAA (Figure 1B). The oxidation state of the radiometals considered ranges from +1 to +4.

### 2.2. Dataset Structure

**Figure 3:**
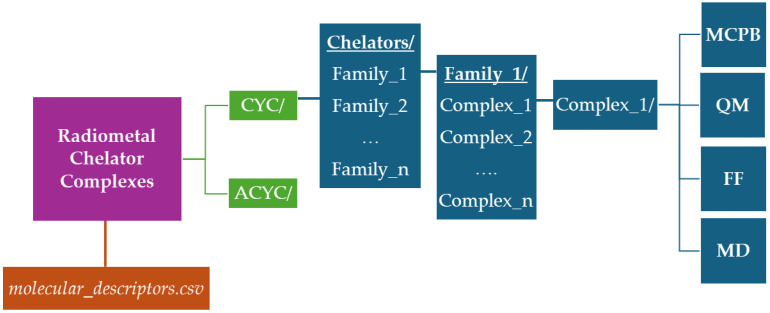
Schematic representation of the radiometal–chelator database structure freely available on figshare [29].

All data generated in this work have been collected into a dataset freely available on figshare [29]. We provide separate directories for cyclic (CYC/) and acyclic (ACYC/) species, including sub-folders corresponding to the different families (e.g., dota/, teta/, …). In turn, each family contains subfolders for the different radiometals, named after the radiometal name and the Cambridge Structural Database ID number (e.g., CYC/dota/ga_782645/). The only exception is represented by the two actinium-based compounds taken from the literature [30] for which the corresponding directories are called CYC/macrodipa/ac_complex and CYC/py2macrodipa/ac_complex. Each compound folder includes a 2D image of the molecular structure (e.g., 2D.png), together with a series of files organized into four subdirectories: MCPB, QM, FF, and MD. Figure 3 depicts a schematic representation of the entire database structure.

#### 2.2.1. Metal Center Parameter Builder Files

For the sake of reproducibility, the MCPB/ folder contains for each compound all files needed to apply the Metal Center Parameter Builder (MCPB) protocol to generate the general AMBER force field parameters (see Section 3). These files include the input MCPB file (mcpb.in), the input experimental structures in .pdb file format, named input_total-charge_spin-multiplicity.pdb (e.g., CYC/dota/ga_782645/input_-1_1.pdb), as well as the .mol2 files of the radiometal (e.g., GA.mol2) and the chelator (LIG.mol2 in all cases).

#### 2.2.2. Quantum-Mechanical Data

QM/ folders contain files associated with QM calculations at the DFT level. In detail, opt.com (opt.log), fc.com (fc.log), and mk.com (mk.log) are the input (output) files of geometry optimization, frequency analysis, and single-point energy calculations, respectively. The formatted Gaussian checkpoint file is also provided (opt.fchk). The final optimized structure is given in .xyz file format following the same name convention as above (e.g., CYC/dota/ga_782645/QM/optimized.xyz). The full list of molecular orbital energies at the optimized geometry is also provided for each molecule (orbital_energies.txt).

#### 2.2.3. Force Field Data

The FF/ folder includes the force field file in .mol2 file format (complex.mol2) containing AMBER atom types, atomic coordinates at the DFT optimized structure, and DFT-based atomic partial charges. The force field modification file with all parameters not included in the general AMBER force field library is also provided (mcpbpy.frcmod). For reproducibility purposes we additionally make available in all cases the AMBER parameter/topology complex_solv.parm7 and coordinate/restart complex_solv.rst7 files used to perform the MD simulation in explicit solvent, as well as the corresponding AMBER-compatible .pdb file complex_solv.pdb.

#### 2.2.4. Molecular Dynamics Data

MD/ folders store the μs-long MD trajectories performed in explicit water solution as a compressed file of 10,000 frames (trajectory.pdb.gz). The representatives of the ten most populated conformational clusters extracted from the trajectory are given in clusters.pdb, and their corresponding population in clusters.dat. From the MD trajectory we determined the statistics of solute-solvent H-bonds, the dynamical evolution of three morphology descriptors related to the gyration tensor (asphericity, acylindricity, and kappa2), and the minimal projection area (data reported in molecular_descriptors.csv).

### 2.3. Sample of Computed Molecular Descriptors

The reported dataset of radiometal–chelator complexes is complemented by the collection of molecular properties computed at different levels of theory. We report standard quantitative-structure relationship parameters derived from the 2D molecular sketch, as well as QM-based and MD-based descriptors. The complete set of computed descriptors (definition and units in Table S3) is available in tabular form (molecular-descriptors.csv) on figshare [29]. In the following, we present and discuss a sample of results from the whole set of computed descriptors.

#### 2.3.1. Computed DFT Energy-Gap as an Indicator of Stability

Experimentally, the stability of a metal–chelator complex can be assessed using its thermodynamic stability constant K_ML_ [17,22], which is associated to the free energy change ∆G occurring when metal (M) and ligand (L) react to give rise to the complex (ML) through the equation: ∆G = −RT Log K_ML_. From a computational perspective, it is common practice to consider the energy gap between the highest occupied molecular orbital (HOMO) and the lowest unoccupied molecular orbital (LUMO) as an indicator of the chemical stability of a molecule [17,31]. Similarly, for open-shell species, the energy gap between the singly occupied molecular orbital (SOMO) and the LUMO is often considered [32]. Although the HOMO/SOMO–LUMO gap reflects the stability of the complex under electronic excitations, and, as such, it has a different physical origin than the thermodynamic metal-ligand stability K_ML_, it represents a computationally cheap and robust way to comparatively characterize different compounds. Therefore, in the present study we followed References [17,31] in which the HOMO–LUMO gap (or, SOMO–LUMO gap for open-shell species) is used as an indicator of stability for metal–chelator complexes.

**Figure 4:**
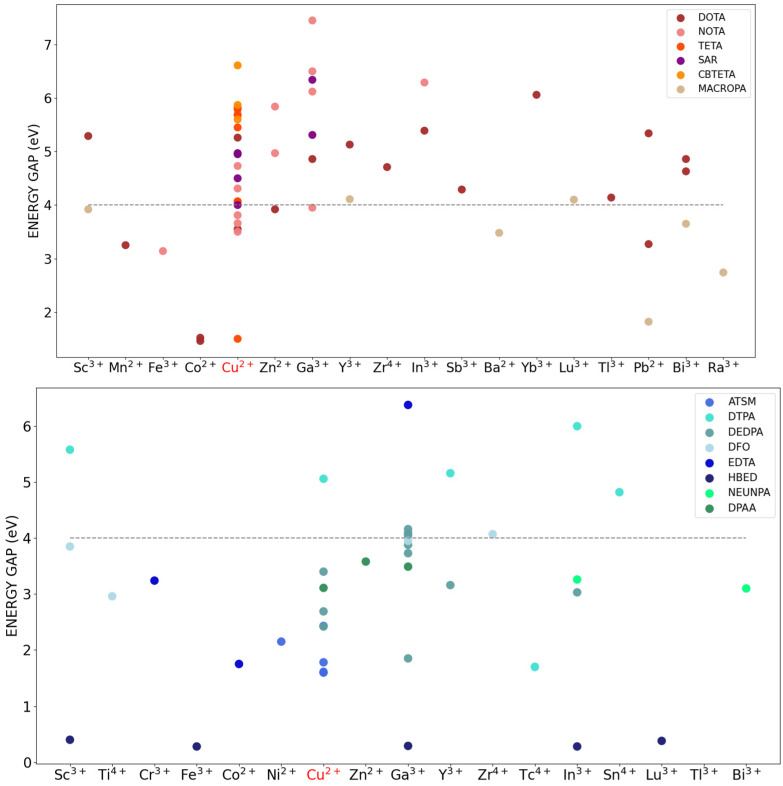
CYC (**top**) and ACYC (**bottom**) chelators–radiometals HOMO/SOMO–LUMO energy gap (eV). Chelators are grouped according to the main families, which also comprise the derivatives. The SOMO–LUMO gap is reported for Cu^2+^ (highlighted in red). The threshold value of stability at 4 eV is highlighted with a dotted line.

We initially checked for both classes of CYC and ACYC compounds and found a good correlation between the available experimental stability constants and the computed energy gap. As an example, for the cyclic chelator NOTA bound to Cu^2+^, Zn^2+^, and Ga^3+^, we found 4.73, 5.84, and 6.50 eV that follow the measured stability constant LogK_ML_ of 21.6, 22.3, and 31.0 kcal/mol [33], respectively (Figure S1A). Similarly, for the acyclic chelator DTPA bound to Y^3+^, Sc^3+^, and In^3+^, we found 4.82, 5.16, and 5.58 eV that follow the measured values of LogK_ML_ of ~22, 27, and 29 kcal/mol [4,34], respectively (Figure S1B). Figure 4 displays the HOMO/SOMO–LUMO energy gap (simply referred to as energy gap) computed for the two series of CYC and ACYC compounds, at increasing atomic number of the radiometal considered. The figure reports only the results for the main chelator families (e.g., DOTA represents all DOTA-bearing CYC complexes, and DTPA all DTPA-bearing ACYC complexes).

On average, a larger value of the energy gap can be observed for CYC compounds (64% above 4 eV as compared to 23% for ACYC ones), and some differences can be found between families. In the case of CYC compounds, higher values are registered for the NOTA, CBTETA, and SAR families (7.45 eV, 6.61 eV, and 6.34 eV, respectively). Interestingly, while SAR-analogue chelators are well-known for their ability to effectively bind copper in terms of both kinetics and thermodynamics [35,36,37], we find an even higher energy gap for the complex with gallium (6.34 eV). Some of the obtained results highlight how small modifications within the same chelator family can affect the stability of the coordination with the same radiometal (Figure S2). As an example, the energy gap of 6.50 eV for the NOTA in complex with Ga^3+^ is found to increase for the derivative carrying phosphonate groups (Ga^3+^-H3NOTP, 7.45 eV) and to decrease for the derivative with phosphonyl propionic acid arms (Ga^3+^-TRAP, 6.12 eV). Overall, lower values of the energy gaps were registered for the MACROPA family (about 4.0 eV on average), regardless of the associated metal ion. This is consistent with the low experimental LogK_ML_ of MACROPA-Pb (16 kcal/mol) [18], which corresponds indeed to the lowest energy gap (1.82 eV). Highlighting the significance of linker groups in the chelation process [38,39,40], addition of phosphonate and carboxylic acid groups to the chelator structure was found to enhance the stability of the complex. For instance, Cu^2+^ complexes with pure DOTA, NOTA, and TETA chelators have lower SOMO–LUMO energy gaps (5.26 eV, 4.73 eV, and 5.68 eV, respectively) compared to derivatives of these chelators with additional functional groups.

For ACYC species, the highest energy gap is registered for the ATSM chelator in complex with Ga^3+^ (6.38 eV), which indeed makes this complex advantageous for applications in radiopharmaceutical chemistry, with respect to other acyclic chelators [41,42]. The DTPA family stands out with the highest energy gaps (e.g., 5.16 eV, 5.58 eV, and 6.00 eV in complex with Y^+3^, Sc^+3^, and In^+3^).

#### 2.3.2. MD-Based Descriptors of Conformation and Solvation

**Figure 5:**
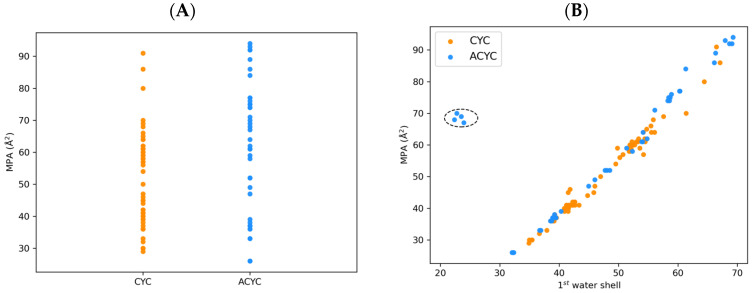
(**A**) Minimal projection area (MPA, Å^2^) of the radiometal–chelator complexes computed during MD simulations. CYC and ACYC chelators are represented as blue and orange dots, respectively. (**B**) Correlation between MPA (Å^2^) and number of water molecules in the first solvation shell of chelator–radiometal complexes computed during MD simulations. The cluster of DFO outliers is highlighted within a dotted circle.

We computed 15 molecular descriptors derived from MD simulations in explicit solvent, by averaging the values of all frames registered along the trajectory. Some of the MD-based descriptors account for the topological and morphological features of compounds (e.g., minimal projection area (MPA) and root mean square fluctuation (RMSF)), while others account for solvation effects (e.g., percentage of H-bonds established with water molecules during MD or number of water molecules in the first and second solvation shells). A physical explanation of these descriptors is provided in Section 3.3. Comparing the differences between morphological descriptors for the CYC and ACYC complexes, we found that, as expected, the latter generally show more extended conformations compared to the former. This behavior is exemplified by the MPA computed for the two classes (Figure 5A). The MPA is defined as the minimum of the circular areas projected perpendicularly to the principal axes of inertia of the molecule, calculated based on the atomic van der Waals radii. Herein, we considered multiple conformers derived from each trajectory frame, and we computed the average value. The more extended the conformations, the higher the value of the MPA. For the ACYC chelators, we obtained higher values with respect to CYC ones (59 ± 21 Å^2^ vs. 50 ± 14 Å^2^, respectively). This can also be observed in Figure 5A, where only three instances of CYC chelators exceeding an MPA value of 70 Å^2^ are reported, while several ACYC ones were found above this value.

We also compared the solvation-related descriptors between the two classes of complexes in combination with the topological ones. We expected a correlation between extended conformations (i.e., high values of MPA) and an increased number of surrounding water molecules in the first solvation shell. Figure 5B shows this correlation for both CYC and ACYC complexes. Interestingly, we found five outliers belonging to the DFO family. The peculiar trend found for this family can be attributed to the high hydrophobicity/aromaticity of these chelators, that can favor intramolecular stacking instead of the interaction with water.

**Figure 6:**
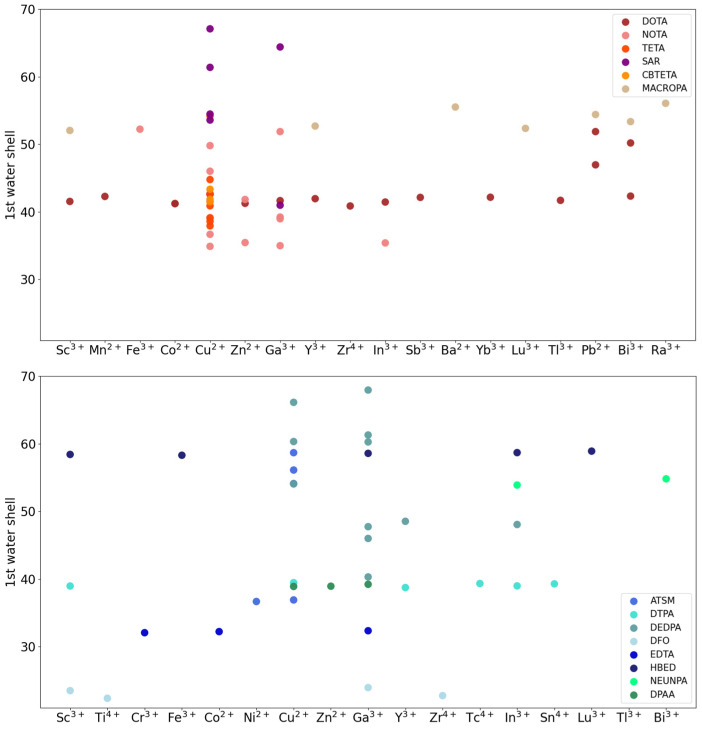
CYC (**top**) and ACYC (**bottom**) chelator–radiometal number of water molecules in the first solvation shell. Chelators are grouped according to the main families, which also comprise the derivatives.

Overall, for both classes we found that, in compounds belonging to the same family, the size of the water shell can vary significantly (e.g., between 41 and 67 for the SAR cyclic family and between 37 and 59 for the ATSM acyclic family). For the same family, small variations are observed with respect to the radiometals loaded. For CYC compounds, the number of water molecules follows approximately the order SAR > MACROPA > DOTA = TETA > CBTETA. Similarly, for ACYC compounds, the following order is observed: HBED > DEDPA > NEUNPA > ATSM > DTPA = DPAA > EDTA > DFO (Figure 6).

## 3. Computational Methods

**Figure 7:**
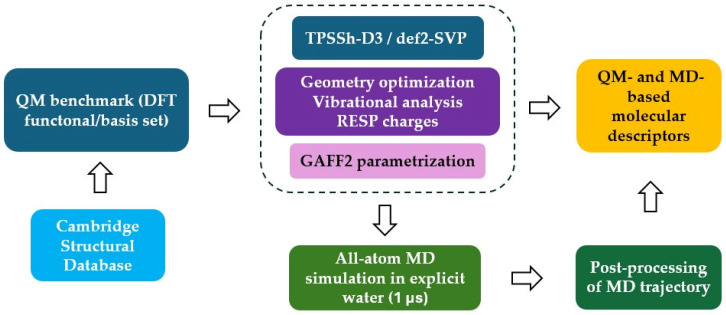
Sketch of the computational protocol adopted in this work.

The overall workflow adopted in the present study is schematically depicted in Figure 7. We downloaded the structures for all complexes from the Cambridge Structural Database [43] except for two actinium-based compounds that were taken from available literature [30]. Starting from these configurations, we performed density functional theory (DFT) geometry optimizations at the TPSSh-D3/def2-SVP level of theory in implicit solvent. This specific combination of exchange-correlation functional and Gaussian basis-set was previously validated on a subset of benchmark structures (Table S4, Figure S3). Frequency calculations and molecular electrostatic potential evaluation at the optimized geometry enabled the derivation of GAFF2 parameters for all compounds. These parameters were used to perform 1 µs-long all-atom MD simulations in explicit water solution for each radiometal–chelator complex.

The 3D structures of radiometal–chelator complexes were downloaded from the Cambridge Structural Database (CSD) [43]. For a few cases for which a given chelator–metal couple was not available in the database, we modified the original structure by manually replacing the desired metal.

### 3.1. QM Calculations

Previous DFT studies on complexes containing transition metals, including those of the *d*- and *f*-blocks, indicate that different flavors of exchange-correlation functionals incorporating some form of dispersion interaction correction provide a good compromise between accuracy and computational cost [30,44,45,46,47,48,49,50,51]. Following these studies, we selected three different functionals accounting for van der Waals interactions, namely the B3LYP-D3 [52], the meta-GGA TPSSh-D3, and the ωB97X-D [53]. The first two are hybrid functionals incorporating empirical atom–atom dispersion corrections [50,51], while the third one belongs to a long-range corrected class of DFT functionals. To expand molecular orbitals, we considered the Karlsruhe basis set def2-SVP for all elements except Ra, Ac, Lu, La, and Yb, for which the all-atom basis set def2-SVP is not available and for which we employed the SDD basis set [54,55]. The performances of the different combinations of functional/basis-set in reproducing available structural data were checked for a sample of a few experimental structures of metal–chelator complexes taken from the Cambridge Structural Database (Figure S3). We performed geometry optimization including the polarizable continuum model with the integral equation formalism variant [56] to mimic the effect of a water solvent. We compared the DFT optimized structure with the experimental one by computing the mean absolute error associated with metal–oxygen and metal–nitrogen bond lengths (Table S4).

The combination TPSSh-D3/def2-SVP exhibited the lowest overall mean MAE value (0.09 Å) as compared to ωB97X-D/def2-SVP and B3LYP-D3/def2-SVP, which show comparable MAE values (0.15 Å and 0.17 Å, respectively). To check if the def2-SVP is adequate, we performed test calculations with the TPSSh-D3 functional by using the def2-TZVP for the metal center and the def2-SVP for the rest of the molecule. While in some specific cases the use of the larger basis set for the metal center yields better agreement with experimental data (e.g., compound 635720), overall, by considering all the benchmark compounds, the improvement appears to be modest (mean values of MAE equal to 0.08 Å and 0.09 Å, respectively, see Table S4). Following these results, systematic QM calculations for the whole sample were performed using the TPSSh-D3/def2-SVP DFT method and the inclusion of an implicit water model. In all cases, geometry optimizations were performed with default self-consistent-field and geometry convergence criteria and without imposing symmetry constraints to the molecule. The final optimized structure and the full set of molecular orbital eigenvalues were obtained using OpenBabel [57] and custom Python scripts.

Geometry optimizations were followed by vibrational frequency calculations, confirming the final conformation to be a global minimum on the potential energy surface (positive eigenvalues in all cases). Reasonable force constants were obtained for all benchmark compounds using the TPSSh-D3/def2-SVP method (Figures S4 and S5). Single-point energy calculations on the optimized geometry were then performed to generate the atomic partial charges fitting the molecular electrostatic potential according to the Merz−Kollman scheme [58] to construct a grid of points around the molecule. Finally, starting from the optimized geometry of each complex, we used the ∆SCF method [59], evaluating total energy differences between the SCF calculations performed for the neutral and charged systems to obtain: (i) the vertical ionization energies and electron affinities (IEv, EAv); and (ii) the quasi-particle corrected HOMO–LUMO gap, usually referred to as the fundamental gap Egap = IE_v_ − EA_v_ = E_N+1_ + E_N−1_ − 2E_N_, (E_N_ being the total energy of the N-electron system). Chemical hardness (η) and softness (S) were obtained as η = Egap/2 and S =1/2η, respectively [60]. All DFT calculations were performed using the Gaussian16 program [61]. To visually check molecular structures, we used GaussView 6.0.

### 3.2. Force Field Parameter Generation

The Gaussian16 outputs of the above three steps (geometry optimization, frequency analysis and single-point energy at the optimized geometry) were used as an input to the Python-based Metal Center Parameter Builder (MCPB.py, [62]). This tool, implemented in the Antechamber package [47,63], has been developed to build force fields for the simulation of metal complexes employing the bonded model approach. In all cases, we applied the Seminario method, a practical procedure to obtain internal force constants from Cartesian second derivatives [64], and the two-step restrained electrostatic potential fitting scheme (RESP [65]) to build the general AMBER force field topology and coordinate files for each radiometal–chelator complex [66]. The reliability of the generated GAFF2 files was validated in all cases by comparing the QM and MM optimized structures (see below).

### 3.3. MD Simulations and Post-Processing

We performed all-atom MD simulations in the presence of an explicit water solution (0.1 M KCl) using the AMBER22 package [67]. We adopted the OPC water model [68] and the corresponding non-bonded models for the monovalent ions [69]. First, each system underwent an energy minimization process that enabled a quick validation of the reliability of the generated GAFF2 parameters. By comparing the QM and MM optimized structures we always found consistent results, with root mean squared displacement (RMSD) in the ranges 0.3–0.7 Å and 0.3–1.3 Å for CYC and ACYC compounds, respectively (Table S5). For small molecules, these low values of RMSD are generally considered indicators of good agreement between different geometries [70]. The minimization step was followed by a brief equilibration to relax the simulation box. In detail, the equilibration was divided into three steps: (1) an annealing of 400 ps in the NVT ensemble, where the temperature was carried from 0 to 400 K and kept constant through the Langevin thermostat; (2) a quenching of 500 ps in the NVT ensemble, where the temperature was carried from 400 to 310 K and kept constant by the Langevin thermostat; and (3) an NPT final equilibration of 1 ns, where pressure and temperature were regulated at 1 atm and 310 K using the isotropic Berendsen barostat and the Langevin thermostat, respectively. Therefore, the whole equilibration phase was 1.9 ns long. The MD production phase was conducted for 1 µs. The simulation was done under the NPT ensemble, with a pressure of 1 atm and a temperature of 310 K. The isotropic Berendsen barostat [71] and the Langevin thermostat [72] were used to control the pressure and temperature, respectively.

Post-processing of MD simulations was performed using the CPPTRAJ program [73]. We computed the atomic root mean square fluctuations (RMSF), the number of waters in the first and second solvation shell, and the formation of intra- and inter-molecular H-bonds. To monitor the different conformations assumed by the molecules along the MD trajectory, the number and population of structural clusters were determined using a hierarchical agglomerative algorithm [74] and the RMSD molecule as a metric. From the MD trajectories we also calculated three morphology descriptors related to the gyration tensor, i.e., asphericity, acylindricity, and kappa2, using custom Python scripts. Asphericity and acylindricity give a measure of the deviation of the mass distribution from spherical and cylindrical symmetry, respectively; the relative shape anisotropy kappa2 is limited between 0 and 1 and reflects both symmetry and dimensionality [28]. The dynamical evolution of all morphological descriptors has been monitored by custom Python scripts. Further details about MD settings and post-processing can be found in our previous publications [25,28].

## 4. Conclusions

In this study, we systematically investigated different molecular features of various radiometal–chelator complexes used in nuclear medicine, through a combination of QM and MD simulations. Given the well-known challenges in modelling transition metals, we aimed to create a reliable set of force field parameters for this class of compounds, which can be re-used for rational design and the development of new radiopharmaceuticals. Furthermore, from both QM calculations and MD simulations, we extracted several key molecular descriptors accounting for complex stability, conformational features, solvation effects, and other electronic- and dynamics-based properties. According to these molecular descriptors, we found peculiar differences between cyclic and acyclic chelators, and between the various families, that can be exploited in future works by machine learning algorithms. Coupling the present data with curated experimental results for a subset of compounds, researchers can leverage our database through supervised machine learning schemes to predict specific properties of the complexes, such as their chemical stability. Additionally, the validated force field parameters make it possible to perform MD simulations of the interaction with biological targets, thus providing additional quantitative features that can be employed in predictive models. The comprehensive dataset created in this work is freely available on figshare [29], providing a reliable computational framework for future investigations on transition metal chelation in radiopharmaceuticals.

## Data Availability

General Amber force field parameters and computed molecular descriptors for the radiometal–chelator complexes investigated in this work are freely available on figshare at 10.6084/m9.figshare.26370964.

## Supplementary Materials

The following supporting information can be downloaded at: https://www.mdpi.com/article/10.3390/molecules29184416/s1, Figure S1: correlation between LogK_ML_ and HOMO–LUMO gap; Figure S2: NOTA and DOTA derivatives in complex with Ga^3+^; Figure S3: 3D sketch of the radiometal–chelator complexes used to perform benchmark DFT calculations; Figure S4: force constants associated to different bonds involving the radiometal in Ga-DOTA as obtained using three different DFT methods with the same def2-SVP basis set; Figure S5: comparison between computed force constant mean values for the benchmark compounds; Table S1: list of radiometals considered in this work; Table S2: list of radiometal–chelator complexes investigated in this work; Table S3: list of computed molecular descriptors; Table S4: mean absolute error obtained at the different levels of DFT for the metal–coordination bond lengths of the benchmark compounds; and Table S5: average RMSDs between the TPSSh-D3/def2-SVP optimized geometry and the minimum-energy structure obtained with the GAFF2 parameters.
