# Supplementary material for: Force Fields, Quantum-Mechanical- and Molecular-Dynamics-Based Descriptors of Radiometal–Chelator Complexes
Source: Molecules. 2024 Sep 17;29(18):4416. doi: 10.3390/molecules29184416 (PMC11434398; doi:10.3390/molecules29184416)
Supplement: Supplementary file 1 [file molecules-29-04416-s001.zip › molecules-3156509-supplementary.pdf]

Force Fields, Quantum-Mechanical- and Molecular-Dynamics-Based Descriptors of Radiometal-Chelator Complexes

Işılay Öztürk, Silvia Gervasoni*, Camilla Guccione, Andrea Bosin, Attilio Vittorio Vargiu, Paolo Ruggerone and Giuliano Malloci*

Department of Physics, University of Cagliari, I-09042, Monserrato (CA), Italy.

*Correspondence: silvia.gervasoni@dsf.unica.it, giuliano.malloci@dsf.unica.it,

SUPPLEMENTARY MATERIALS

Figure 1 IUPAC names:

NOTA = 1,4,7-triazacyclononane-1,4,7-triacetic acid
DOTA = 1,4,7,10-tetraazacyclododecane-1,4,7,10-tetraacetic acid
TETA = 1,4,8,11-tetraazacyclotetradecane-1,4,8,11-tetraacetic acid
CB-TETA = 1,4,8,11-Tetraazabicyclo[6.6.2]hexadecane-4,11-diacetic acid
SAR = 3,7,11-trimethyl-2,6,10-trioxo-1,5,9-trioxa-3,7,11-trisiladodecane (Sarcophagine)
MACROPA = 6-[1,4,7,10-tetraazacyclododecane-1-yl]-N,N',N''-tris[(1,1 dimethylethyl)phenylphosphinyl]-1,3,5-triazine-2,4-diamine
DTPA = 1,1,4,7,7-diethylenetriaminepentaacetic acid
DFO= N'-[5-(acetyl hydroxylamine)pentyl]-N-[5-(acetyl hydroxyamino)-5-oxo pentyl]-N''-hydroxybutanediamide
DEDPA=1,2-[{6-(carboxylato)pyridin-2-yl}methylamino]-Ethane
EDTA = Ethylenediaminetetraacetic acid
ATSM = Diacetyl bis(N(4)-methylthiosemicarbazonato
HBED = N,N′-bis(2-hydroxy benzyl)ethylenediamine-N,N′-diacetic acid
NEUNPA = N,N'-Bis(2-hydroxy benzyl)ethylenediamine-N,N'-diacetic acid
DPAA = N,N'-bis(2-aminoethyl)-2,2'-diamino-diacetic acid

Supplementary Figures

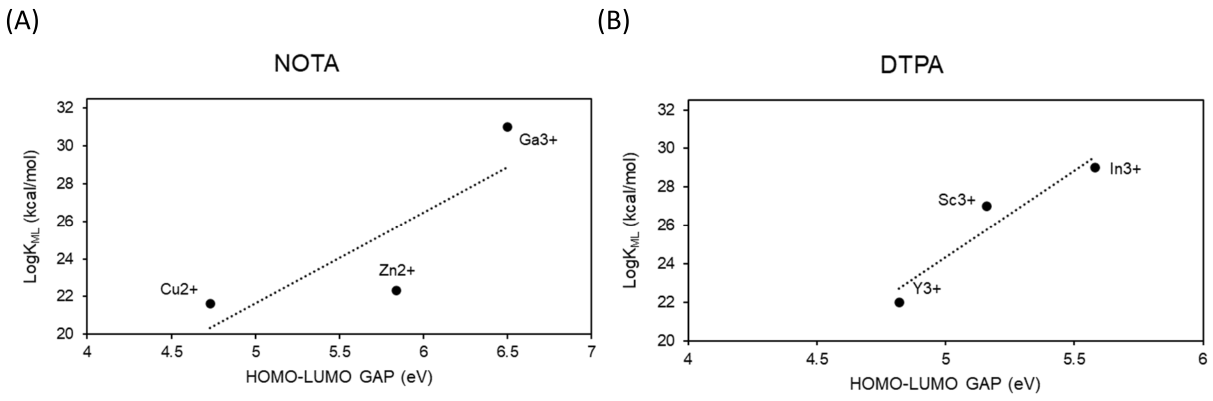

Figure S1. Correlation between LogK$_{ML}$ and HOMO-LUMO gap in two examples: (A) NOTA (CYC) and (B) DTPA (ACYC). Each point represents a chelator-radionuclide complex. The dotted lines indicate the good linear correlation between the two quantities.

NOTA H3NOTP TRAP

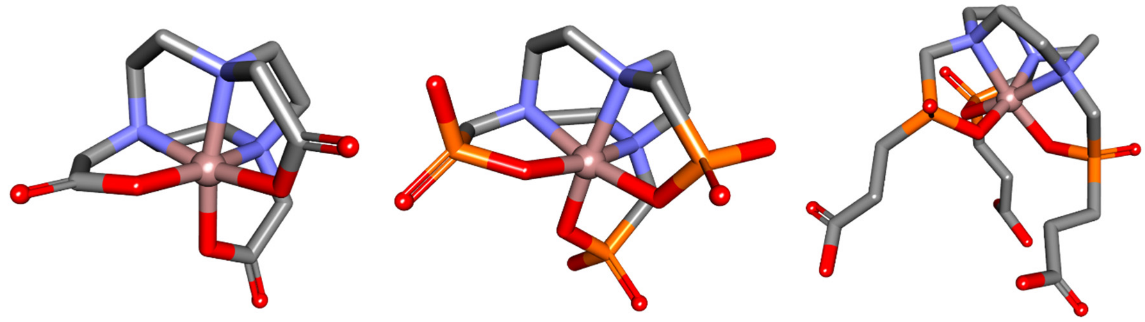

Figure S2. NOTA and its derivatives (H3NOTP and TRAP), in complex with Ga^{3+} (pink sphere). Hydrogen atoms are omitted for clarity. Carbon, nitrogen and oxygen atoms are colored in gray, blue, and red, respectively.

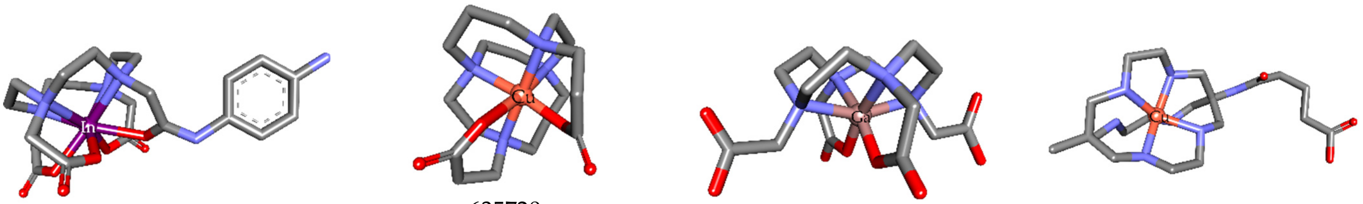

228929
In-DOTA

635720
Cu-CBTETA

606649
Ga-DOTA

915824
Cu-SAR

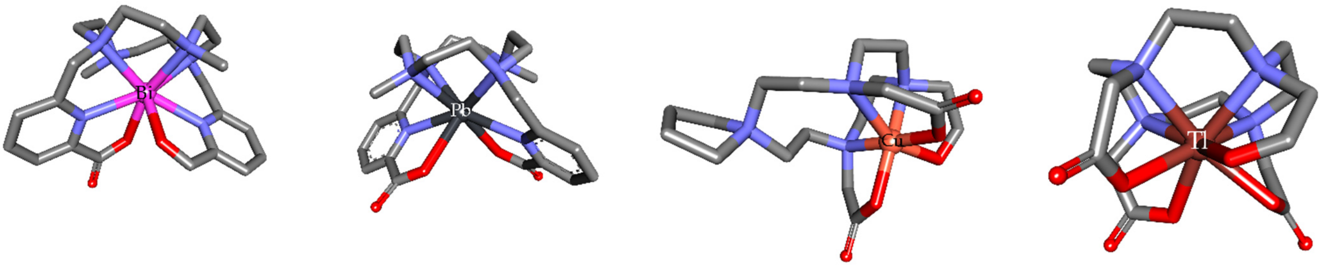

1009076
Bi-Me-do2pa

1049922
Pb-Me-do2pa

2171219
Cu-HDO3A

2171219
Tl-DOTA

Figure S3. 3D sketch of the radiometal-chelator complexes (with corresponding CSD numbers) used to perform benchmark DFT calculations. Hydrogen atoms are omitted for clarity. Carbon, nitrogen and oxygen atoms are colored in gray, blue, and red, respectively.

BOND	B3LYP-D3	ω-B97X-D	TPSSh-D3
Ga-O1	104.7	115.5	105.4
Ga-O2	105.2	117	106.1
Ga-N1	11.5	10.7	19.2
Ga-N2	12.1	8.3	22.1
Ga-N3	13.3	12.4	22.2
Ga-N4	33.5	37.6	39.3
**mean value**	46.72	50.25	52.38

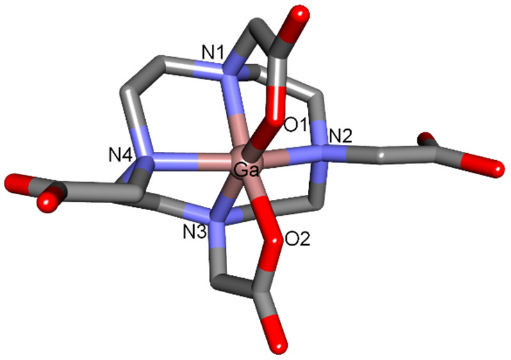

Figure S4. Force-constants (kcal/mol/Å^2) associated to different bonds involving the radiometal in Ga-DOTA (CSD 606649) as obtained using three different DFT methods (B3LYP-D3, ω-B97X-D, TPSSh-D3) with the same def2-SVP basis set.

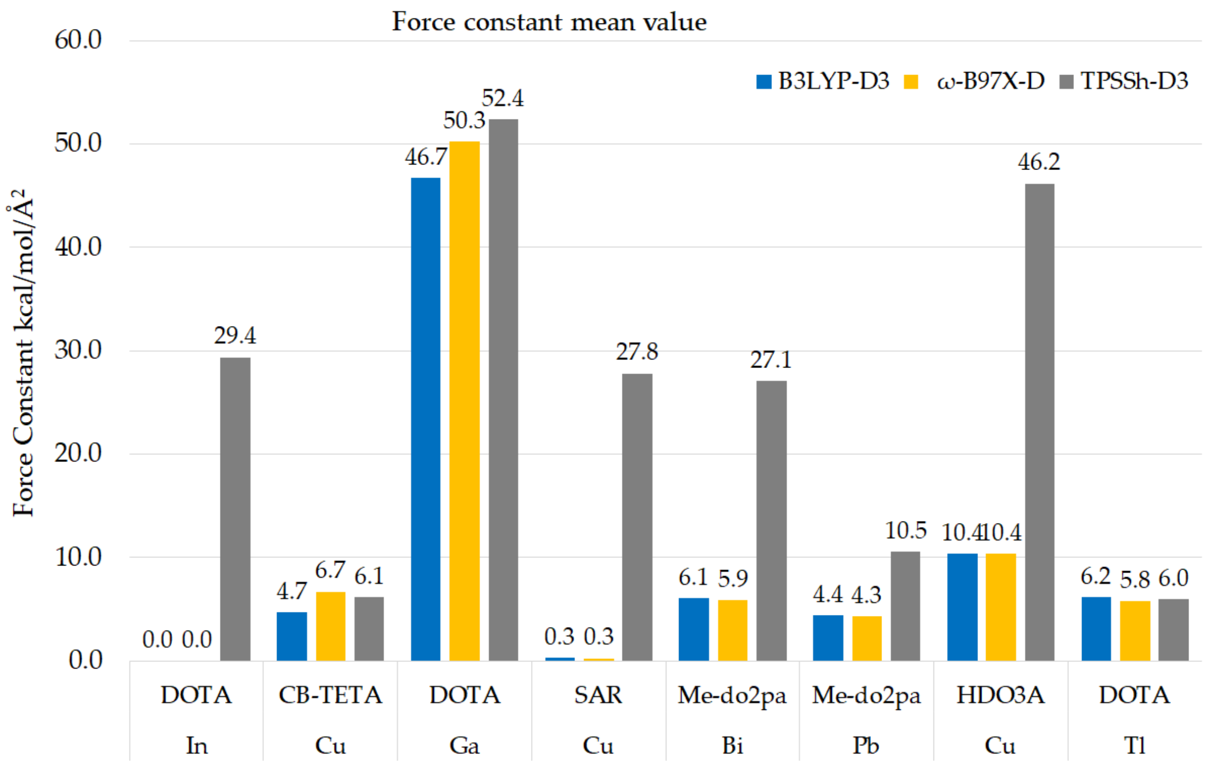

Figure S5. Comparison between computed force-constants mean values for the benchmark compounds shown in Figure S3, as obtained with three different DFT functionals in combination with the Gaussian def2-SVP basis set.

Supplementary Tables

Radionuclide	Decay mode	Application	Chelators
Sc^{3+}	β-	Therapy	DOTA, MACROPA, MACRODIPA, PYMACRODIPA, **DTPA, HBED, DFO**
Mn^{2+}	β+	PET	NOTA, DOTA, **DTPA, EDTA**
Fe^{3+}	β-	Imagining	NOTA, **HBED**
Co^{2+}	EC	Imagining	NOTA, DOTA
Cu^{2+}	β+/β-	Theranostics	DOTA, NOTA, TETA, SAR, **DTPA, DEDPA, ATSM, BISPA**
Zn^{2+}	β+	PET	NOTA, DOTA, **EDTA**
Ga^{3+}	ɣ/β+	PET	DOTA, NOTA
Y^{3+}	β-	PET	MACROPA, **DTPA, OCTAPA**
Tc^{4+}	ɣ	SPECT	NOTA, **DTPA, EDTA**
Zr^{4+}	β+	PET	DOTA, **DFO, EDTA**
In^{3+}	ɣ	SPET	NOTA, DOTA, **DTPA, EDTA, HBED, DEDPA**
Sb^{3+}	β+	SPECT	DOTA
Ba^{2+}	β-		MACROPA
Tl^{3+}	ɣ	SPECT	DOTA, **PYPA**
Pb^{2+}	β-	PET	DOTAM, DO2PA, NOON-2Py, Crown-4Py
Bi^{3+}	α/β-	Therapy	DOTA, DO2PA, MACROPA, MACRODIPA, PYMACRODIPA, **NEUNPA**
Ra^{2+}	α	Therapy	DOTA, MACRODIPA
Ac^{3+}	α	Alpha Therapy	MACRODIPA
Yb^{3+}	β-		DOTA
Lu^{3+}	β-	Therapy	DOTA, MACROPA, **HBED**
Ti^{4+}	β+	PET	**DFO**
Cr^{3+}	ɣ		**EDTA**
Ni^{2+}	β+/β-	PET	**ATSM**
Sn^{4+}	ɣ	Threapy	**DTPA**

Table S1. List of radionuclides considered in this work and, their main decay channels and chelators (acyclic chelators in boldface).

					General Properties	
CSD Identifier	CSD Number	Radiometal	Total Charge	Number of Atom	Chemical Formula	Molecular Weight (g/mol)
			DOTA ANALOGUES			
DARSUO	2128467	Bi^{3+}	-1	53	C16H24N4O8Bi	609.36
ZAGSAF	1846451	Bi^{3+}	3	69	C18H38N10O2Bi	635.54
QEZWIB	160465	Co^{2+}	-2	53	C16H24N4O8Co	459.32
AZANEW	1505012	Cu^{2+}	-2	53	C16H24N4O8Cu	463.93
AMAJEE	782645	Ga^{3+}	-1	53	C16H24N4O8Ga	470.11
AQEJEL	228929	In^{3+}	-1	53	C16H24N4O8In	515.20
HONYUF	718992	Mn^{2+}	-2	53	C16H24N4O8Mn	455.32
BABQOO	2022138	Pb^{2+}	2	61	C16H32N8O4Pb	607.68
ZAGSAF	1846451	Pb^{2+}	2	69	C18H38N10O2Pb	633.76
DARSOI	2128466	Sb^{3+}	-1	53	C16H24N4O8Sb	522.14
JOGZEM	984842	Sc^{3+}	-1	53	C16H24N4O8Sc	445.34
ONELEA	1025647	Tl^{3+}	-1	53	C16H24N4O8Tl	604.77
HODCEI	101669	Y^{3+}	-1	53	C16H24N4O8Y	489.29
AGINUB	933967	Yb^{3+}	0	56	C16H28N5O6Yb	559.46
QEZWIB	160465	Zn^{2+}	-2	53	C16H24N4O8Zn	465.79
UCITOS	1501174	Zr^{4+}	0	53	C16 H24 N4O8Zr	491.60
NOJFUQ	1577991	Co^{2+}	2	53	C14H28N6O4Co	403.34
KEDYAV	876185	Cu^{2+}	2	48	C15H25N5O2Cu	370.94
			NOTA ANALOGUES			
SUVTEK01	1876232	Cu^{2+}	2	52	C18H24N6S3Cu	484.16
GIVQEK	1876235	Cu^{2+}	2	61	C22H28N6O2S2Cu	536.17
XACVOQ	2020766	Cu^{2+}	1	39	C13H19N4O2Cu	326.86
XACVUW	2020767	Cu^{2+}	0	44	C15H20N4O4Cu	383.89
BEGCOJ	2116477	Cu^{2+}	0	54	C16H28N4O5Cu	419.96
AWENEV	219829	Cu^{2+}	-1	40	C12H18N3O6Cu	363.83
MUYJAS	749080	Fe^{3+}	0	70	C18H33N3O12P3Fe	632.23
JAYTIM	1183521	Ga^{3+}	0	40	C12H18N3O6Ga	370.01
RAVXOF	2081615	Ga^{3+}	0	46	C9H21N3O9P3Ga	477.92
VIQVEA	2256758	Ga^{3+}	1	44	C15H20N4O4Ga	390.07
MUYJEW	749081	Ga^{3+}	0	70	C18H33N3O12P3Ga	646.11
JAYTIM	1183521	In^{3+}	0	40	C12H18N3O6In	415.11
JAYTIM	1183521	Zn^{2+}	-1	40	C12H18N3O6Zn	365.70
GIVPUZ	1876233	Zn^{2+}	2	52	C18H24N6S3Zn	486.00
			TETA ANALOGUES			
PARQIJ	1228363	Cu^{2+}	0	47	C14H24N4O4Cu	375.91
MEMYUY	148563	Cu^{2+}	0	49	C14H26N4O4Cu	377.93
KEYHUS	624742	Cu^{2+}	-2	59	C18H28N4O8Cu	491.98
KEYJEE	624744	Cu^{2+}	2	49	C14H28N4O2Cu	347.94
CIDWES	635720	Cu^{2+}	0	59	C18H32N4O4Cu	432.02
NIRMIL	671044	Cu^{2+}	1	55	C16H30N5O3Cu	403.99
CUXGUZ	902630	Cu^{2+}	-2	67	C22 H35 N5 O4Cu	497.09

			SAR ANALOGUES			
BALPAI	1036058	Cu^{2+}	2	87	C26H44N8O7SCu	676.29
SAPBES	861491	Cu^{2+}	0	77	C22H40N8O6Cu	576.15
	861491	Ga^{3+}	3	85	C24H46N8O6Ga	612.40
DINWII	915824	Cu^{2+}	2	72	C20H41N7O3Cu	491.13
DINWII	915824	Cu^{2+}	2	71	C19H40N8O3Cu	492.12
PAYXEU	886231	Ga^{3+}	4	58	C14H35N8Ga	385.20
			CBTETA ANALOGUES			
ZAGJID	1053276	Cu^{2+}	0	52	C12H28N4O5P2Cu	433.87
RUNNIA	1975657	Cu^{2+}	0	57	C14H30N4O6P2Cu	475.90
WAVTUK	843734	Cu^{2+}	0	55	C15H29N4O5PCu	439.93
			MACRO ANALOGUES			
			MACROPA-DIPA ANALOGUES			
	*	Ac^{3+}	1	73	C26H34N4O8Ac	757.57
LAWXAM	2124116	Bi^{3+}	1	73	C26H34N4O8Bi	739.55
LAWXAM	2124116	Sc^{3+}	1	73	C26H34N4O8Sc	575.53
			MACROPA ANALOGUES			
BEDJAY	1569022	Lu3+	1	73	C26H34N4O8Lu	705.54
BEDJAY	1569022	Pb^{2+}	0	73	C26H34N4O8Pb	737.77
BEDJAY	1569022	Ra^{3+}	0	73	C26H34N4O8Ra	756.57
BEDJAY	1569022	Sc^{3+}	1	73	C26H34N4O8Sc	575.53
BEDJAY	1569022	Y^{3+}	1	73	C26H34N4O8Y	619.48
BEDJAY	1569022	Ba^{2+}	0	73	C26H34N4O8Ba	667.90
BEDJAY	1569022	Bi^{3+}	1	73	C26H34N4O8Bi	739.55
			NOON-2Py ANALOGUES			
HEFLAJ	2160769	Pb^{2+}	2	69	C24H36N4O4Pb	651.77
			PYMACRODIPA ANALOGUES			
	*	Ac^{3+}	1	75	C29H33N5O7Ac	790.60
LAWXEQ	2124117	Bi^{3+}	1	75	C29H33N5O7Bi	772.58
LAWXEQ	2124117	Sc^{3+}	1	75	C29H33N5O7Sc	608.56
			CROWN-4Py ANALOGUES			
HEFKUC	2160767	Pb^{2+}	2	95	C36H48N8O2Pb	832.02
			OTHERS			
			DO2A2S ANALOGUES			
AZAGAM	2078038	Cu^{2+}	0	63	C18H34N4O4S2Cu	498.16
			DO2PA ANALOGUES			
HOQKUV	1009076	Bi^{3+}	1	67	C24H32N6O4Bi	677.53
AJIQUH	1049922	Pb^{2+}	0	67	C24H32N6O4Pb	675.75
			DO3A ANALOGUES			
ZESWON	2171218	Cu^{2+}	-1	48	C14H23N4O6Cu	406.90
ZESROI	2171219	Cu^{2+}	0	59	C18H30N4O6Cu	462.00
JEQHIA	2204293	Ga^{3+}	0	69	C23H30N5O10Ga	606.23
			ATSM ANALOGUES			
DOFCOS	1001048	Cu^{2+}	1	56	C21H26N7SCu	472.09
UMUXUV	213899	Cu^{2+}	0	31	C8H14N6S2Cu	321.91
TEWRUM	2205695	Cu^{2+}	0	56	C19H24N10S2Cu	520.14

ERABEF	830440	Cu^{2+}	0	51	C14H25N7O2S2Cu	451.07
UMUXOP	213898	Ni^{2+}	0	31	C8H14N6S2Ni	317.06
			AZAPA ANALOGUES			
GENZUW	919037	Cu^{2+}	0	85	C36H34N10O4Cu	734.27
GEPBAG	919038	Ga^{3+}	1	85	C36H34N10O4Ga	740.44
GEPBAG	919038	In^{3+}	1	85	C36H34N10O4In	785.54
			DEDPA ANALOGUES			
IMADOR	1458998	Cu^{2+}	0	77	C28H30N10O8Cu	698.15
GIDWUP	2218829	Cu^{2+}	2	65	C22H32N6O4Cu	508.07
GIDXAW	2218830	Cu^{2+}	0	87	C38H34N6O8Cu	766.26
POXQOK	1055008	Ga^{3+}	1	51	C20H22N4O4Ga	452.14
POXQUQ	1055009	Ga^{3+}	-1	61	C24H24N4O8Ga	566.19
MUHCOJ	1404246	Ga^{3+}	1	71	C26H26N10O8Ga	676.27
GIDXEA	2218831	Ga^{3+}	1	87	C38H34N6O8Ga	772.43
OSUDOW	833686	Ga^{3+}	1	41	C16H16N4O4Ga	398.05
OSUDUC	833687	Ga^{3+}	1	73	C30H26N6O8Ga	668.29
POXQOK	1055008	In^{3+}	-1	55	C22H20N4O8In	583.23
POXQOK	1055008	Y^{3+}	-1	55	C22H20N4O8Y	557.32
			DFO ANALOGUES			
HINGUK	2262707	Sc^{3+}	0	55	C21H24N3O6Sc	459.39
GAVZIM	1164388	Ga^{3+}	0	55	C21H24N3O6Ga	484.15
BEJNEN	2156705	Ti^{4+}	1	55	C21H24N3O6Ti	462.30
GAVZIM	1164388	Zr^{4+}	1	55	C21H24N3O6Zr	505.66
			DPAA ANALOGUES			
GEJLEP	1530704	Cu^{2+}	-1	38	C16H12N3O6Cu	405.83
GEJLEP	1530704	Ga^{3+}	0	38	C16H12N3O6Ga	412.01
GEJLEP	1530704	Zn^{2+}	-1	38	C16H12N3O6Zn	407.69
			DPAADAB ANALOGUES			
GEJNER	1530703	Ga^{3+}	-1	46	C19H15N3O8Ga	483.06
			DTPA ANALOGUES			
ETPACU01	1150570	Cu^{2+}	-3	46	C14H18N3O10Cu	451.85
MOQVOD	1213339	In^{3+}	-2	46	C14H18N3O10In	503.12
MOMLAD	984843	Sc^{3+}	-2	46	C14H18N3O10Sc	433.26
KIJWUV01	1196767	Sn^{2+}	-3	46	C14H18N3O10Sn	507.02
KIJWUV02	1196767	Sn^{4+}	-1	46	C14H18N3O10Sn	507.02
MOQVOD	1213339	Tc^{4+}	-1	46	C14H18N3O10Tc	486.31
ABUPET	253287	Y^{3+}	-2	46	C14H18N3O10Y	477.21
			EDTA ANALOGUES			
BALCUQ	2049762	Co^{2+}	-2	33	C10H12N2O8Co	347.14
BALCUQ	2049762	Cr^{3+}	-1	33	C10H12N2O8Cr	340.21
FADJIE	182842	Ga^{3+}	-1	33	C10H12N2O8Ga	357.93
			HBED ANALOGUES			
ACACAM	2076262	Fe^{3+}	-1	69	C30H28N4O6Fe	596.41
ACACEQ	2076263	Ga^{3+}	-1	69	C30H28N4O6Ga	610.29
ACACEQ	2076263	In^{3+}	-1	69	C30H28N4O6In	655.38
ACACEQ	2076263	Lu^{3+}	-1	69	C30H28N4O6Lu	715.53

ACACEQ	2076263	Sc^{3+}	-1	69	C30H28N4O6Sc	585.52
			NEUNPA ANALOGUES			
BEXYEM	2141221	Bi^{3+}	0	68	C24H29N6O8Bi	738.50
BEXYEM	2141221	In^{3+}	0	68	C24H29N6O8Bi	738.50
			OTHERS			
			BISPOX ANALOGUES			
NOGVOX	1908811	In^{3+}	1	91	C41N6O7In	803.29
			Bn2DT3A ANALOGUES			
QEVLUC	1864389	Ga^{3+}	0	62	C24H28N3O6Ga	524.22
			BPC ANALOGUES			
AHAPEE	184959**	In^{3+}	0	51	C24H16N6O4In	567.24
			PYPA ANALOGUES			
QEXMOZ	2115641	Tl^{3+}	-1	60	C25H21N5O8Tl	723.85

Table S2. List of radiometal-chelator complexes investigated in this work. Input structures of actinium complexes are taken from [30]. ** Cobalt was replaced with indium based on recent research indicating BPCA as a promising new chelator for indium [4].

Name	Unit	Description
CSD		Cambridge Data Base identifier
Name		IUPAC name
Radiometal		Radiometal symbol and oxidation state
Total Charge	e	Total molecular charge
Number of Atom		Total number of atoms
Formula		Molecular formula
ChiralAtms		Number of atoms bonded to four different groups on structure
FlexTorsion		The flexible rotational angles around single bonds within a molecule
Mass	g/mol	Molecular weight of the molecule
HbAcc		Hydrogen bond acceptor
HbDon		Hydrogen bond donor
HOMO	eV	Energy associated to the highest occupied molecular orbital
LUMO	eV	Energy associated to the lowest unoccupied molecular orbital
HL GAP	eV	HOMO-LUMO energy gap
IP	eV	Ionization Potential
EA	eV	Electron Affinity
Electronegativity		Electronegativity
Chem hardness	eV	Chemical hardness
Chem softness	eV	Chemical softness
ROT_A	GHz	Rotational constant A associated to the optimized geometry
ROT_B	GHz	Rotational constant B associated to the optimized geometry
ROT_C	GHz	Rotational constant C associated to the optimized geometry
dipole_moment	Db	Molecular dipole moment associated to the optimized geometry
H bond	%	Solute-solvent H-bond interactions
1st water shell		Average number of water molecules in the 1st solvation shell
2nd water shell		Average number and of water molecules in the 2nd solvation shell
RMSF ± SD	Å	Average value and standard deviation of the RMSF of atomic positions in MD trajectory
MPA ± SD	$Å^2$	Average value and standard deviation of the minimal projection area associated to the configurations explored by the molecule during MD trajectory
Asphericity ± SD		Average asphericity and standard deviation computed along MD. This parameter gives a measure of the deviation of the mass distribution from spherical symmetry
Acylindricity ± SD		Average acylindricity and standard deviation computed along MD. This parameter gives a measure of the deviation of the mass distribution from cylindrical symmetry
KAPPA2 ± SD		Average relative shape anisotropy kappa2 and standard deviation computed along MD This parameter reflects both symmetry and dimensionality of the molecule

Table S3. List of computed molecular descriptors, corresponding units and detailed definition.

	MAE (Å)			
**CCDC**	**B3LYP-D3/ def2-SVP**	**TPSSh-D3/ def2-SVP**	**TPSSh-D3/ Def2-SVP//def2-TZVP**	**ωB97X-D/ def2-SVP**
228929	0.07	0.05	0.07	0.06
635720	0.28	0.25	0.08	0.30
606649	0.05	0.07	0.04	0.04
915824	0.13	0.04	0.13	0.04
1009076	0.09	0.07	0.06	0.08
1049922	0.15	0.07	0.12	0.13
2171219	0.50	0.07	0.05	0.45
1025647	0.08	0.07	0.07	0.07
**Mean Value of MAE**	**0.17**	**0.09**	**0.08**	**0.15**

Table S4. Mean absolute error (MAE) obtained at the different levels of DFT for both the metal-O and metal-N coordination bond lengths of the selected benchmark compounds (see Figure S3). The MAE was computed with respect to the experimental starting geometries, downloaded from the CSD.

Cyclic Chelators Analogues	Mean ± STD
DOTA	0.35 ± 0.17
NOTA	0.40 ± 0.34
TETA	0.36 ± 0.17
CBTETA	0.25 ± 0.07
SAR	0.72 ± 0.36
MACROPA	0.36 ± 0.12
**Acyclic Chelators Analogues**	**Mean ± STD**
ATSM	0.64 ± 0.15
AZAPA	1.25 ± 0.15
DEDPA	0.69 ± 0.38
DFO	0.48 ± 0.16
DPAA	0.60 ± 0.26
DTPA	0.52 ± 0.11
EDTA	0.41 ± 0.06
HBED	0.60 ± 0.21
NEUNPA	0.31 ± 0.05

Table S5. Average RMSDs and standard deviations (Å) between the heavy atoms of the TPSSh-D3/def2-SVP optimized geometry and the minimum-energy structure for each complex obtained with the MCBP.py and GAFF2 parameters of the database.
